# Inhibition of the Phospholipase Cε–c-Jun N-Terminal Kinase Axis Suppresses Glioma Stem Cell Properties

**DOI:** 10.3390/ijms23158785

**Published:** 2022-08-07

**Authors:** Masashi Okada, Yurika Nakagawa-Saito, Yuta Mitobe, Asuka Sugai, Keita Togashi, Shuhei Suzuki, Chifumi Kitanaka

**Affiliations:** 1 Department of Molecular Cancer Science, School of Medicine, Yamagata University, 2-2-2 Iida-Nishi, Yamagata 990-9585, Japan; 2 Department of Neurosurgery, School of Medicine, Yamagata University, 2-2-2 Iida-Nishi, Yamagata 990-9585, Japan; 3 Department of Ophthalmology and Visual Sciences, School of Medicine, Yamagata University, 2-2-2 Iida-Nishi, Yamagata 990-9585, Japan; 4 Department of Clinical Oncology, School of Medicine, Yamagata University, 2-2-2 Iida-Nishi, Yamagata 990-9585, Japan; 5 Research Institute for Promotion of Medical Sciences, Faculty of Medicine, Yamagata University, 2-2-2 Iida-Nishi, Yamagata 990-9585, Japan

**Keywords:** glioma initiating cell, brain tumor initiating cell, phospholipase Cε, c-Jun N-terminal kinase

## Abstract

Glioma stem cells (GSCs), the cancer stem cells of glioblastoma multiforme (GBM), contribute to the malignancy of GBM due to their resistance to therapy and tumorigenic potential; therefore, the development of GSC-targeted therapies is urgently needed to improve the poor prognosis of GBM patients. The molecular mechanisms maintaining GSCs need to be elucidated in more detail for the development of GSC-targeted therapy. In comparison with patient-derived GSCs and their differentiated counterparts, we herein demonstrated for the first time that phospholipase C (PLC)ε was highly expressed in GSCs, in contrast to other PLC isoforms. A broad-spectrum PLC inhibitor suppressed the viability of GSCs, but not their stemness. Nevertheless, the knockdown of PLCε suppressed the survival of GSCs and induced cell death. The stem cell capacity of residual viable cells was also suppressed. Moreover, the survival of mice that were transplanted with PLCε knockdown-GSCs was longer than the control group. PLCε maintained the stemness of GSCs via the activation of JNK. The present study demonstrated for the first time that PLCε plays a critical role in maintaining the survival, stemness, and tumor initiation capacity of GSCs. Our study suggested that PLCε is a promising anti-GSC therapeutic target.

## 1. Introduction

Cancer stem cells (CSCs) are a small population of cells in tumor tissue that exhibit both resistance to therapies and tumor initiation potential, and, thus, remain after treatment and cause recurrence. The nature and vulnerabilities of CSCs need to be elucidated in more detail because their elimination may improve patient survival. We previously reported the molecular mechanisms and signaling pathways that are involved in maintaining the stemness of glioma stem cells (GSCs), which are the CSCs of glioblastoma [1,2,3,4,5]. Since the molecular mechanisms underlying the regulation of GSCs remain largely unknown, further studies on the factors contributing to the survival and maintenance of GSC stemness are warranted.

Phosphoinositides account for only a small fraction of phospholipids; however, their levels are regulated by various lipid kinases, phosphatases, and phospholipases in response to different external stimuli, and the dysregulation of phosphoinositide metabolism has been implicated in a number of diseases, including cancer [6,7,8]. Phosphoinositide-specific phospholipase C (PI-PLC), one of the phosphoinositide metabolic enzymes, is an enzyme family of 13 isozymes that are classified into six subfamilies, which hydrolyze phosphatidylinositol (4,5)-bisphosphate (PtdIns (4,5) P2) to produce two second messengers, diacylglycerol (DAG) and inositol 1,4,5-trisphosphate (InsP3) [6,9]. PtdIns (4,5) P2 is involved in cell adhesion and migration, and InsP3, one of the products of the hydrolysis of PtdIns (4,5) P2 by PLC, promotes Ca^2+^ release from the endoplasmic reticulum, while the other product, DAG, mediates the activation of PKC. These signal transducers play important roles in the regulation of cancer cell migration, proliferation, and death [6,8]. In addition to the regulation of PtdIns (4,5) P2 levels by PLC activity, several PLCs are involved in a number of intracellular signaling pathways due to their subfamily-specific domain structures, and the activity and expression of PLCs in various tumors differ from those in normal tissue [6,10]. However, the role of PLCs in cancer remains unclear and controversial and has rarely been reported in CSCs.

The present study revealed that the expression of PLCε, a member of the PLC family, was higher in GSCs than in genetically identical (isogenic) differentiated GSCs and was essential not only for the survival of GSCs, but also the maintenance of their stemness. Therefore, we propose PLCε as a promising therapeutic target for GSC-targeted therapy.

## 2. Results

### 2.1. The Knockdown of PLCε Suppresses the Viability of GSCs

**Figure 1:**
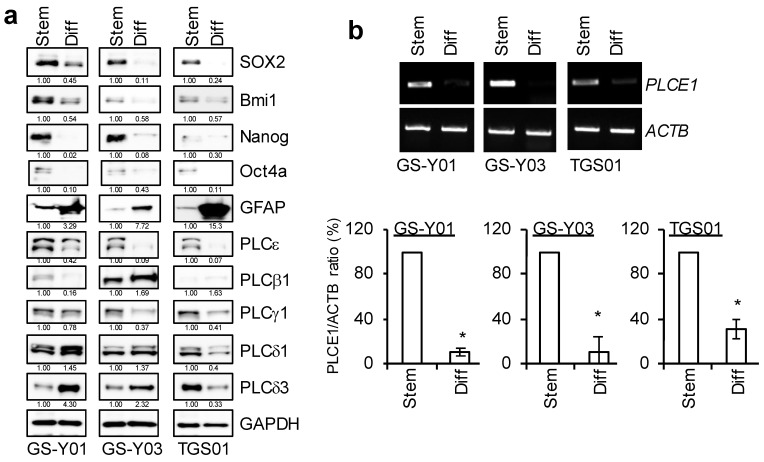
**PLCε is highly expressed in glioma stem cells (GSCs), but not in differentiated GSCs.** GSCs (GS-Y01, GS-Y03, and TGS01; Stem) and isogenic differentiated GSCs (Diff) were evaluated by immunoblot analyses (**a**) or RT-PCR (**b**) for the indicated proteins and mRNAs. Representative images of two (**a**) or three (**b**) biological replicates are shown. The numbers below the Western blot images show the relative band intensities after each band was quantified by densitometry and normalized by the GAPDH value. Graphs indicate the quantification of PCR analyses by densitometry. The values are presented as the means ± SDs of the triplicate samples of an independent experiment. * *p* < 0.05 vs. Stem by the Student’s *t*-test.

We previously reported that three independent cells (GS-Y01, GS-Y03, and TGS01) that were isolated from patients with glioblastoma possessed glioma stem-like properties when they were cultured under specific conditions without serum, and that the differentiation of these three patient-derived GSCs was induced when they were cultured as an adherent monolayer in medium containing serum [1,11,12]. Since the high expression of PLCs has been shown to correlate with malignancy in a number of tumors [13,14,15,16,17,18,19], we attempted to compare the expression patterns of various PLCs between GSCs and isogenic differentiated GSCs. Similar to our previous findings, Western blotting results showed that the expression of SOX2, Nanog, and Bmi1, markers of stem cell properties, was higher in GSCs and lower in isogenic differentiated GSCs, while the expression of GFAP, a differentiation marker, was inversely correlated (Figure 1a). We then examined the expression levels of various PLCs. The results that were obtained showed that the expression of PLCε was high in all GSCs that were examined in the present study and decreased with differentiation, while other PLCs showed no consistent changes. PLCε was also more abundantly expressed in GSCs than in isogenic differentiated GSCs at the mRNA level (Figure 1a,b).

**Figure 2:**
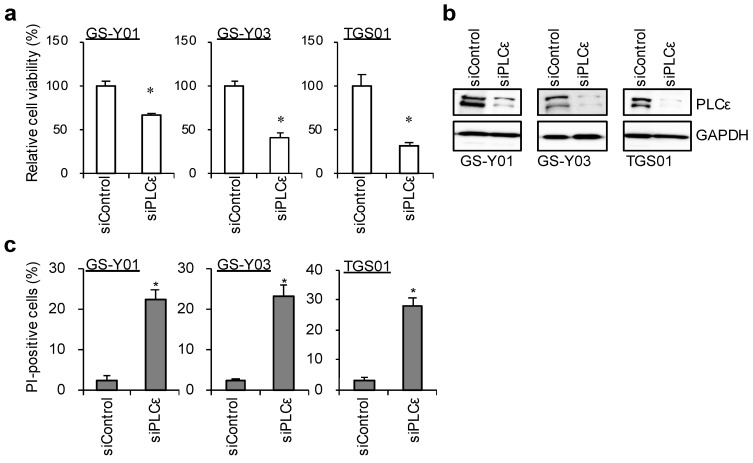
**Genetic silencing of PLCε suppresses cell viability.** GSCs (GS-Y01, GS-Y03, and TGS01) were transiently transfected with siRNA against PLCE1. The cells were then subjected to a cell viability assay using WST-8 (**a**), Western blot analyses (**b**), or a propidium iodide (PI) uptake assay (**c**). The values represent the means + SDs of the triplicate samples of a representative experiment. Similar results were obtained from two independent biological replicates. * *p* < 0.05 vs. siControl-transfected cells by the Student’s *t*-test. Representative fluorescence images of are shown at Supplemental Figure S2.

These results indicate that PLCε is highly expressed among the various PLC isoforms in these GSCs. We performed a knockdown analysis to verify the function of PLCε, which is highly expressed in GSCs. The transient knockdown of PLCε significantly suppressed its expression in GSCs (Figure 2b). Since the inhibition of viability was observed, we examined the viability of GSCs following the knockdown of PLCε using the water-soluble tetrazolium (WST) assay. The survival rates of the three GSCs that were tested decreased after the knockdown of PLCε (Figure 2a). We then investigated whether the knockdown of PLCε induced cell death in GSCs. To verify the induction of cell death by the knockdown of PLCε, the propidium iodide (PI) uptake assay was performed on cells after the transient knockdown of PLCε. The results that were obtained showed that the knockdown of PLCε significantly induced cell death in the three GSCs that were tested (Figure 2c). These results indicate that the expression of PLCε is important for the survival of GSCs.

### 2.2. PLCε Contributes to the Maintenance of GSC Stemness

**Figure 3:**
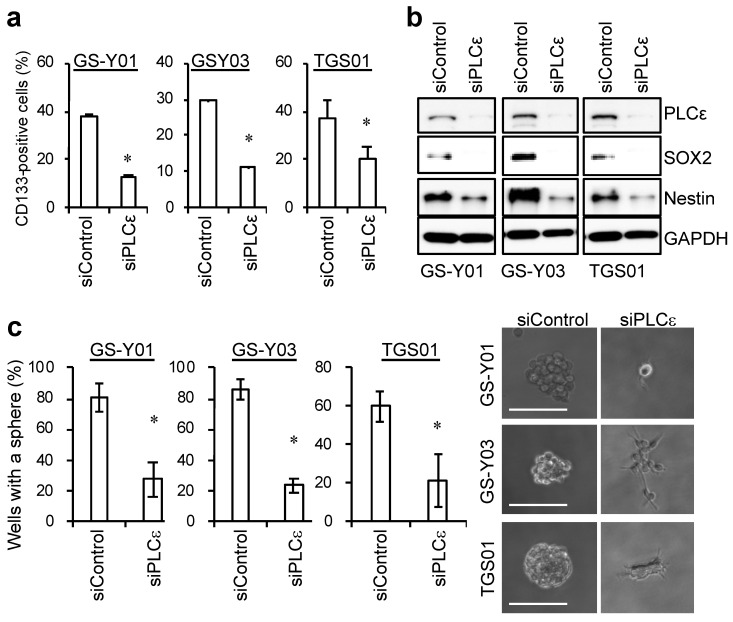
**Genetic silencing of PLCε by siRNA suppresses the stemness of GSCs.** (**a**) GSCs were transiently transfected with siRNA against PLCE1 or control siRNA. After 4 days, the transfected cells were subjected to flow cytometric analyses for the cell surface expression of CD133. The graphs show the means + SDs of triplicate samples. Representative flow cytometric histograms are shown at Supplemental Figure S3. (**b**) Cells that were transfected as in (**a**) were subjected to Western blot analyses for the expression of the indicated proteins. (**c**) Cells that were transfected as in (**a**) were subjected to sphere-forming analyses. Graphs show the means ± SDs of triplicate samples (left). Representative photographs of spheres are shown (right). Bars: 100 μm. Similar results were obtained from two independent biological replicates. * *p* < 0.05 vs. siControl-transfected cells by the Student’s *t*-test.

Since the knockdown of PLCε in GSCs significantly induced cell death, we examined the effects of the knockdown of PLCε on the self-renewal capacity of surviving GSCs. To achieve this, we examined changes in the expression of stem cell markers with the knockdown of PLCε. siRNA against PLCε was transfected into GSCs, and the living cells were gated 96 h later to test the expression of the stem cell marker, cell surface CD133. The percentatge of CD133-positive cells significantly decreased in the fractions of viable GSCs following the transient knockdown of PLCε (Figure 3a). In addition, the expression levels of other stem cell markers, SOX2 and Nestin, were decreased by the knockdown of PLCε (Figure 3b). Since the knockdown of PLCε suppressed the expression of stem cell markers in GSCs, we investigated whether it also inhibited the sphere-forming ability of GSCs, an indicator of their self-renewal capacity. A sphere-forming assay using viable cells that were transiently transfected with siRNA against PLCε showed that the number of GSCs forming spheres was significantly lower in PLCε-knockdown GSCs than in the control GSCs (Figure 3c). These results indicate that PLCε plays a pivotal role in maintaining the stemness of GSCs.

### 2.3. PLCε Contributes to the Tumor-Initiating Capacity of GSCs

**Figure 4:**
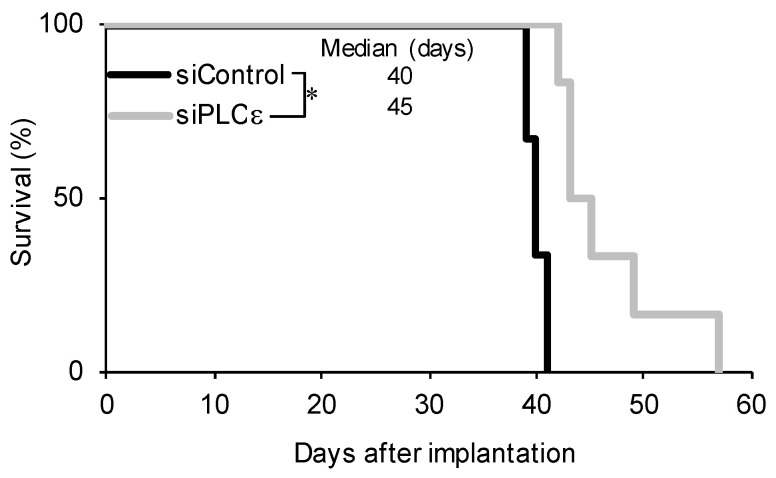
**Silencing of PLCε prolongs survival in the xenograft model.** GS-Y03 cells were transiently transfected with siRNA against PLCE1 or siControl for 4 days, after which the cell viability was assessed. An equal number (1 × 10^4^) of viable cells was then implanted intracranially into each nude mouse. A Kaplan–Meier analysis shows the survival curves for mice (*n* = 6 for each group). * *p* < 0.05 vs. siControl-transfected cells by the Log-rank test.

Since the suppression of PLCε expression not only had a negative impact on the survival of GSCs, but also inhibited the self-renewal capacity of surviving GSCs, we investigated whether another important feature of GSCs, tumor-initiating capacity, was lost in surviving GSCs. To achieve this, GSCs that were transfected with siRNA were implanted intracranially into immunodeficient mice. All the mice that were implanted with cells that were transfected with control siRNA developed brain tumors after approximately 40 days and died quickly thereafter (Figure 4). In contrast, the implantation of viable cells that were transiently transfected with siRNA against PLCε eventually resulted in the formation of brain tumors in all the recipient mice; however, survival was significantly longer than that in the control group (Figure 4). This result suggested that the transient knockdown of PLCε reduced the number of tumor-initiating cells. Collectively, these results suggest that PLCε plays a critical role in maintaining the self-renewal and tumor-initiating potential of GSCs.

### 2.4. Down-Regulation of PLCε Suppresses the Stemness of GSCs by Inhibiting the JNK Axis

**Figure 5:**
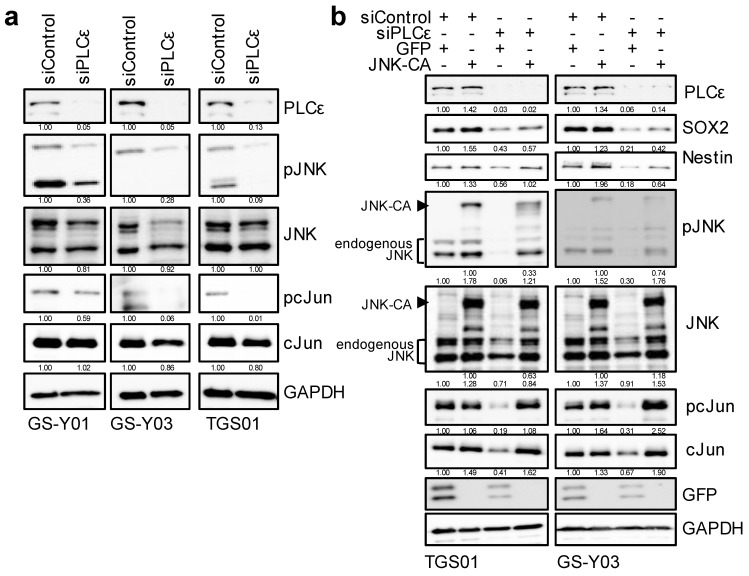
**PLCε silencing suppresses GSC stemness by inhibiting the JNK axis.** (**a**) GSCs (GS-Y01, GS-Y03, and TGS01) transiently transfected with siRNA against PLCE1 were subjected to Western blot analyses for the expression of the indicated proteins. (**b**) TGS01 and GS-Y03 cells that were transiently transfected with the activated JNK1 expression plasmid (JNK-CA) or control vector (GFP) for 24 h were transiently transfected with siRNA against PLCE1 or control siRNA for 4 days. The indicated proteins were detected by Western blotting. Similar results were obtained from two independent biological replicates. The numbers below the Western blot images show the relative band intensities after each band was quantified by densitometry and normalized by the GAPDH value.

Since PLCε was shown to play an essential role in maintaining the stemness of GSCs, we attempted to elucidate the molecular mechanisms underlying the maintenance of GSC stemness by PLCε. We previously reported that the activation of the JNK axis was essential for maintaining the stemness of various CSCs, including GSCs [1,20,21]. Therefore, we investigated whether the knockdown of PLCε affected JNK signaling in GSCs. We found that the knockdown of PLCε inhibited JNK signaling (pJNK and pc-Jun) in GSCs (Figure 5a). To clarify whether the reduction that was observed in the stemness of GSCs that was due to the down-regulation of PLCε was mediated by JNK signaling, we expressed constitutively active JNK, the MEK-JNK fusion protein, and then induced the knockdown of PLCε. The results that were obtained revealed that the pre-transfection of this constitutively active JNK fusion protein into GSCs partially canceled the reduction in stemness that was caused by the knockdown of PLCε (Figure 5b). These results suggest that the maintenance of the stem cell capacity of GSCs by PLCε is mediated by JNK.

## 3. Discussion

PLCε is one of the PI-PLC family of 13 isozymes, and in contrast to other PLC isoforms, it has not only a lipase catalytic domain, but also a Ras/MAPK pathway activation domain; therefore, it functions as an important signaling hub that regulates various biological cellular processes [22,23,24]. The present results revealed that the expression of PLCε was higher in GSCs than in isogenic differentiated GSCs and that its high expression contributed to the survival of GSCs and the maintenance of stemness via the activation of JNK.

To the best of our knowledge, PLCε has not been implicated in the survival or maintenance of glioma cells/GSCs. In addition, the role of PLCε in human cancers has been widely examined but remains highly controversial. Previous studies reported that the expression of PLCε was up-regulated in esophageal squamous cell carcinoma, renal cell carcinoma, and bladder cancer, correlating with tumor invasiveness and decreased patient survival, while its suppression inhibited the growth of and induced cell death in these cells [25,26,27]. Furthermore, the high expression of PLCε was shown to enhance tumorigenicity [28,29]. However, the molecular mechanisms by which PLCε affects cell proliferation and tumorigenesis remain unclear. In a two-step skin carcinogenesis model with 7,12-dimethylbenz (a)anthracene and the phorbol ester, 12-O-tetradecanoylphorbol-13-acetate (TPA), the knockout of PLCε affected carcinogenic resistance and suppressed chronic inflammation that was induced by TPA [30,31]. The expression of CCL2/MCP1, IL-22, and IL-23 was previously shown to be enhanced in transgenic mice in which PLCε was overexpressed in skin keratinocytes due to the activation of NFκB [32,33]. These findings suggest that continually high expression levels of PLCε contribute to tumor initiation by inducing chronic inflammation through the sustained triggering of inflammatory signals. Since the expression of CCL2/MCP1 was shown to be higher in glioblastoma than in non-tumor tissue [34], and the NFκB and JNK pathways, well-known inflammatory signals, were found to be activated in GSCs [1,35], it is likely that the sustained activation of inflammatory signaling via high PLCε expression levels plays an important role in the initiation of glioblastoma. Recent studies reported that the activation of JNK was associated with tumorigenesis and tumor growth in various types of carcinomas [36,37,38]. The activation of JNK is known to up-regulate the expression of inflammatory cytokines (IL-1, IL-6, and TNF-α) and chemokines (CCL2 and CCL5), leading to chronic inflammation and tumor initiation [39]; therefore, JNK, a representative inflammatory kinase, is strongly suggested to be one of the key players in tumor initiation. Although further studies are needed to elucidate the molecular mechanisms underlying the up-regulation of PLCε in GSCs, the present results suggest that high PLCε expression levels in GSCs induce the activation of JNK, which may be involved in maintaining the stemness in GSCs via the activation of inflammatory signals. Therefore, PLCε has potential as a molecular target for GSC-targeted therapy. In this regard, it is interesting to note that shPLCε suppressed JNK activity by decreasing the expression of MEK4 in prostate cancer, thereby reducing the expression of twist, a transcription factor that strongly induces epithelial–mesenchymal transition [40]. In contrast, in non-small cell lung cancers with a mutant Ras-dependent carcinogenic mechanism, PLCε was shown to inhibit cell proliferation by inducing the down-regulation of PLCε upon oncogenic K-ras expression [41]. Since oncogenic Ras mutations, such as those that were observed in pancreatic and lung cancers, are very rare in glioblastoma, and their expression induces autophagic cell death, it may be difficult for PLCε to act as a tumor suppressor in glioblastoma [42,43]. However, further studies are required to elucidate the detailed functions of PLCε in maintaining CSCs, in which PLCε has been shown to exert tumor suppressive effects.

The present study revealed that among the 13 isozymes of PLCs, PLCε was important for the maintenance of GSCs. There are currently no selective inhibitors for PLCε. In our experiments with U73122, a commonly used broad-spectrum PLC activity inhibitor, the suppression of broad PLC activity inhibited the cell viability of some GSCs, even at concentrations that did not affect the normal fibroblast cell line, IMR90, which is consistent with previous findings that were obtained using various tumor cells (Supplemental Figure S1A) [44,45]. However, it did not induce consistent changes in the expression patterns of stem cell and differentiation markers in GSCs (Supplemental Figure S1B) and did not markedly suppress the sphere-forming ability of GSCs, a measure of their self-renewal capacity (Supplemental Figure S1C). Collectively, these results suggest that the inhibition of broad-spectrum PLC activity did not effectively suppress the stemness of GSCs, in contrast to the knockdown of PLCε. One possible reason for the failure of U73122 to suppress the stemness of GSCs is that signaling pathways are complex due to the presence of isozymes with various domain structures in PLCs [6,10] and isozymes may function antagonistically with each other. Nevertheless, since PtdIns (4,5) P2, a PLC substrate, localizes at cleavage furrows and focal adhesions and its metabolism is essential for the regulation of biological activities, such as cell division, migration, and adhesion [46,47,48], PLC activity may still be necessary for the biological functions of active CSCs in small residual lesions after therapy or in metastases. Preclinical studies on several small molecule PLC inhibitors are currently underway [49]. If, although we have yet to obtain direct evidence, the PLC activity of PLCε is specifically required for the maintenance of cancer stemness, and also if the suppression of the activity of other PLCs with U73122 functions antagonistically against the suppression of stemness, novel PLC inhibitors that selectively inhibit PLCε may be promising therapeutic agents.

## 4. Materials and Methods

### 4.1. Antibodies and Chemicals

Antibodies against PLCε (07-513), PLCγ1 (05-366), Bmi1 (05-637), and Nestin (MAB5326) were purchased from Merck Millipore (Billerica, MA, USA). An antibody against SOX2 (MAB2018) was purchased from R&D Systems Inc. (Minneapolis, MN, USA). Antibodies against GFAP (#3670), Nanog (#4903), phospho-JNK (#4668), phosphor-c-Jun (#2361), OCT-4A (#2890), GAPDH (#5174), were purchased from Cell Signaling Technology Inc. (Beverly, MA, USA). Antibodies against PLCβ1 (sc-205), PLCδ3 (sc-514912), and GFP (sc-9996) were purchased from Santa Cruz Biotechnology, Inc. (Santa Cruz, CA, USA). Anti-PLCδ1 (610356) was purchased from BD Biosciences (Franklin Lakes, NJ, USA). Anti-CD133 (W6B3C1) was purchased from Miltenyi Biotech (Bergisch Gladbach, Germany). U73122 (U6756) was purchased from Merck Millipore.

### 4.2. Cell Culture

Isolation, establishment, and characterization of stem-like properties of patient-derived GSCs (GS-Y01, and GS-Y03; previously designated #38) were conducted as previously described [50,51]. TGS01 was a generous gift from the Department of Neurosurgery, University of Tokyo. The characterization of TGS01 has been described elsewhere [52]. The GSCs were maintained under previously reported monolayer stem cell culture conditions [1,12]. The differentiation of GSCs was induced by culturing cells in DMEM/F-12 medium that was supplemented with 10% fetal bovine serum (FBS, Thermo Fisher Scientific, Waltham, MA, USA), 100 units/mL of penicillin, and 100 μg/mL of streptomycin for 2 weeks [1,12]. IMR90, a human normal fetal lung fibroblast cell line, was obtained from the American Type Culture Collection (Manassas, VA, USA) and maintained in DMEM that was supplemented with 10% FBS. All IMR90 experiments were performed using cells with a low passage number (<8).

### 4.3. WST-8 Assay

We conducted the WST-8 assay to assess cell viability [11,12]. Cells (1–5 × 10^3^/well) that were plated on 96-well collagen I-coated plates were treated with drugs, as described in the figure legends. WST-8 reagent (Cell Counting Kit-8, DOJINDO LABORATORIES, Kumamoto, Japan) was then added and the cells were incubated at 37 °C for 1–2 h. Absorbance at 450 nm was measured using a microplate reader (iMark; Bio-Rad, Hercules, CA, USA). The relative cell viability was calculated as a percentage of the absorbance of the treated samples relative to that of the controls.

### 4.4. Propidium Iodide (PI) Uptake Assay

The propidium iodide uptake assay was used to assess cell death [53,54]. In brief, the cells were incubated with PI (1 μg/mL) and Hoechst33342 (10 μg/mL) at 37 °C for 5 min. To calculate the ratio of PI-positive cells (dead cells) to Hoechst-positive cells (total cells), fluorescent images were obtained using a fluorescence microscope (CKX41; Olympus, Tokyo, Japan) and scored. More than 250 cells were counted to calculate the percentage of PI-positive cells.

### 4.5. Western Blotting

A Western blot analysis was conducted as previously described [12,55]. The cells were harvested after the removal of medium and washed with ice-cold phosphate-buffered saline (PBS), and then lysed in RIPA buffer (10 mM Tris/HCl (pH 7.4), 0.1% sodium dodecyl sulfate (SDS), 0.1% sodium deoxycholate, 1% Nonidet P-40, 150 mM NaCl, 1 mM EDTA, 1.5 mM sodium orthovanadate, 10 mM sodium fluoride, 10 mM sodium pyrophosphate, and protease inhibitor cocktail set III (FUJIFILM Wako Chemicals, Osaka, Japan)). The lysate was immediately mixed with the same volume of 2 × Laemmli buffer (125 mM Tris/HCl (pH 6.8), 4% SDS, and 10% glycerol) and boiled at 95 °C for 10 min. The protein concentrations of the cell lysates were measured using a BCA protein assay kit (Pierce Biotechnology, Inc., Rockford, IL, USA). The samples containing equal amounts of protein were separated by SDS/polyacrylamide gel electrophoresis and transferred to polyvinylidene difluoride membranes. The membranes were probed with the indicated primary antibodies and appropriate HRP-conjugated secondary antibodies, as recommended by the manufacturer of each antibody. Regarding the reprobing of immunoblots, antibodies were stripped from the probed membrane using stripping buffer (2% SDS, 100 mM β-mercaptoethanol, and 62.5 mM Tris-HCl (pH 6.8)). After stripping, the membranes were washed with Tris-buffered saline with Tween 20 and blocked with skim milk. The membranes were then reprobed with appropriate antibodies. Immunoreactive bands were visualized using Immobilon Western Chemiluminescent HRP Substrate (Merck Millipore) and detected by a ChemiDoc Touch device (Bio-Rad). Quantification of the bands in the gels was performed by densitometry using ImageJ software (version 1.53k) (http://imagej.nih.gov/ij/).

### 4.6. Flow Cytometric Analysis

A flow cytometric analysis was conducted as previously described [21,54]. In analyses of CD133 expression, dissociated cells were washed with PBS, fixed with 4% (*w*/*v*) paraformaldehyde at room temperature (RT) for 10 min, and washed again with PBS. The cells were blocked in FCM buffer (0.5% [*w*/*v*] bovine serum albumin and 0.1% [*w*/*v*] NaN_3_ in PBS) for 1 h, followed by three PBS rinses, a further incubation with the anti-CD133 antibody in FCM buffer at 4 °C overnight, and then an incubation with Alexa Fluor^®^ 488 goat anti-mouse IgG at RT for 1 h. The cells exhibiting a signal for CD133 above the gate established by the isotype control were considered to be positive for CD133. At least 1 × 10^4^ cells were evaluated and gated using side and forward scatters to identify viable cell populations. All flow cytometry experiments were run on the FACSCanto^TM^ II flow cytometer (BD Biosciences, Franklin Lakes, NJ, USA) and the data were analyzed using FlowJo software, version 7.6.5 (FlowJo LLC, Ashland, OR, USA).

### 4.7. Reverse Transcription–PCR Analysis

Total RNA was extracted from cells using Trizol (Thermo Fisher Scientific) and 1 µg of the total RNA was reverse transcribed using the PrimeScript RT reagent kit (Takara Bio Inc., Shiga, Japan) according to the manufacturer’s protocol. The target genes were amplified with Quick Taq HS DyeMix (Toyobo CO, LTD, Osaka, Japan). A RT-PCR analysis was performed using the following primers: *PLCE1* (forward: 5′-CGCTGTGGAGTTGTTTGGTG, reverse: 5′-AAGCACTGGATGGGCTCTTG) and *ACTB* (forward: 5′-CCCATGCCATCCTGCGTCTG, reverse: 5′-CGTCATACTCCTGCTTGCTG). Quantification of the bands in the gels was performed by densitometry using ImageJ software (version 1.53k).

### 4.8. Sphere-Forming Analysis

The sphere-forming assay was performed as previously described [21,54,56,57]. Cells in the monolayer culture were dissociated, and the cells were serially diluted in the stem cell culture medium and then seeded onto non-coated 96-well plates such that each well contained a single cell. The wells containing a single cell were marked under a phase-contrast microscope the next day, and the percentage of marked wells with a sphere relative to the total number of marked wells was calculated 7–10 days after seeding. We counted spheres with a diameter ≥ 50 μm.

### 4.9. Animal Study

Mouse xenograft studies were performed as previously described [1,21]. Regarding intracranial implantation, 6–9-week-old male BALB/cAJcl-*nu/nu* mice (CLEA Japan Inc., Tokyo, Japan) were anesthetized with medetomidine, midazolam, and butorphanol (0.3, 4, and 5 mg per kg body weight, respectively) before viable cells that were suspended in 5 μL of PBS were injected stereotactically into the right corpus striatum (2.5 mm anterior and 2.5 mm lateral to the bregma, and 3.0 mm deep). After implantation, the general health status of and the appearance of neurological symptoms in the recipient mice were monitored. All the animal experiments were performed following a protocol that was approved by the Animal Research Committee of Yamagata University.

### 4.10. Gene Silencing by siRNA

siRNAs against human PLCε (PLCE1; HSS121828, 121829, and 181915) and medium GC duplex #2 of Stealth RNAi^TM^ siRNA negative control duplexes were purchased from Thermo Fisher Scientific. The cells were transfected with a mixture of the 3 different siRNAs against PLCE1 (siPLCε; total 200–250 pmol per 6-cm dish) or with control siRNA (siControl; 200–250 pmol per 6-cm dish) using Lipofectamine RNAiMAX (Thermo Fisher Scientific) according to the manufacturer’s instructions.

### 4.11. Statistical Analysis

All data are shown as the means + standard deviations. The data were analyzed using the Student’s *t*-test for comparisons between two groups. Mouse survival was evaluated by the Kaplan–Meier method and analyzed using the Log-rank test. Differences with a *p*-value < 0.05 were considered to be significant and are indicated with asterisks in the figures.

## 5. Conclusions

In conclusion, a comparison between GSCs and differentiated GSCs revealed that the expression of PLCε was higher in the GSCs and also that PLCε contributed to the survival and maintenance of GSC stemness. Moreover, we demonstrated that at least one of the molecular mechanisms maintaining stemness by the high expression of PLCε in GSCs involved the activation of JNK (Supplemental Figure S4). The present study is the first to report the potential of PLCε as a therapeutic target in GSCs, which represents an important milestone in PI-PLC and GSC research.

## Data Availability

Not applicable.

## Supplementary Materials

The following supporting information can be downloaded at: https://www.mdpi.com/article/10.3390/ijms23158785/s1.
