# Supplementary material for: Inhibition of the Phospholipase Cε–c-Jun N-Terminal Kinase Axis Suppresses Glioma Stem Cell Properties
Source: Int J Mol Sci. 2022 Aug 7;23(15):8785. doi: 10.3390/ijms23158785 (PMC9369372; doi:10.3390/ijms23158785)
Supplement: Supplementary file 1 [file ijms-23-08785-s001.zip › ijms-1837039-supplementary.pdf]

Supplemental Figure S1

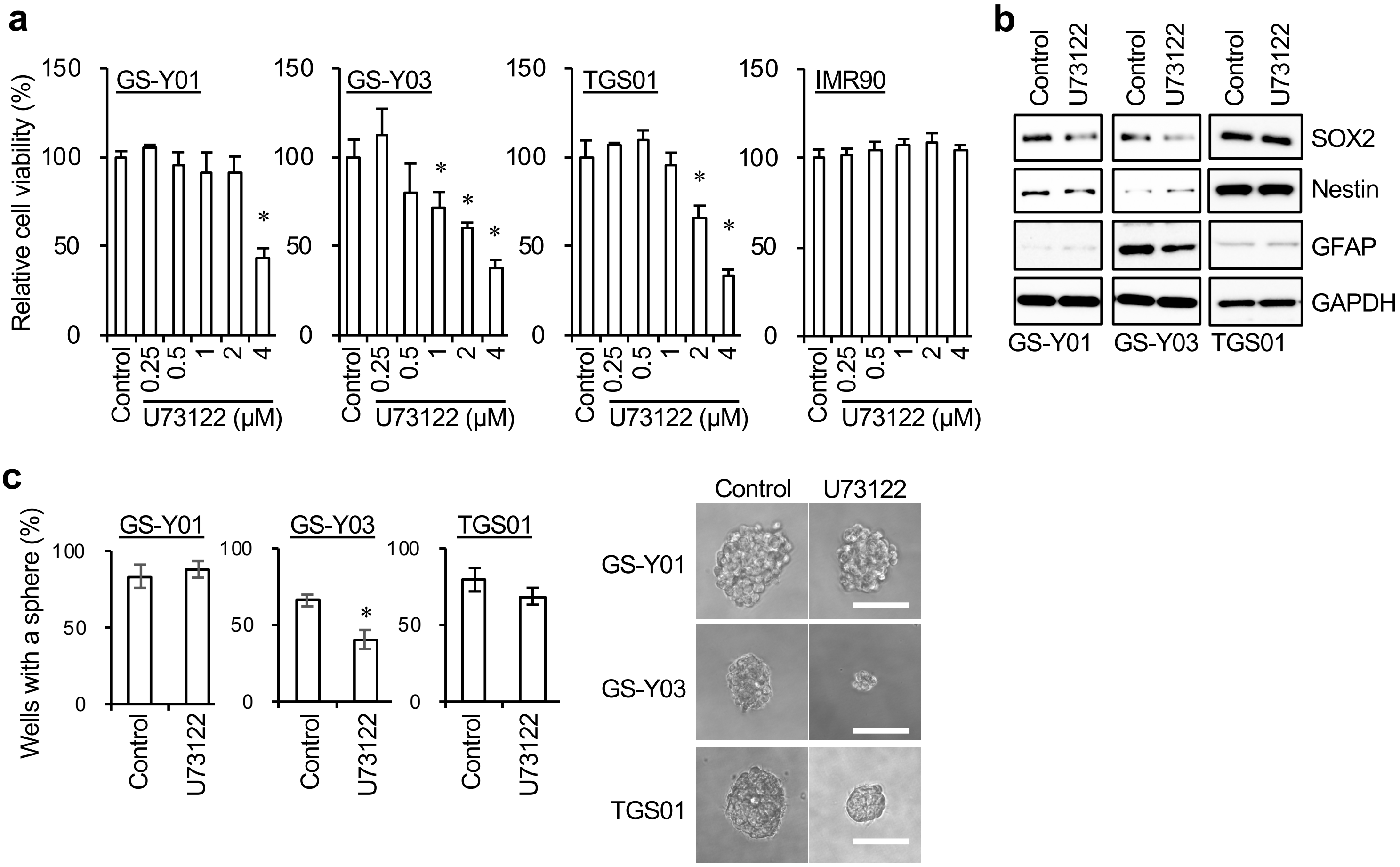

Figure S1. An inhibitor of PI-PLC activity reduces the cell viability, but not the stemness of GSCs. (a) GSCs (GS-Y01, GS-Y03, and TGS01) and IMR90 cells treated with U73122 at the indicated concentrations were subjected to a cell viability assay using WST-8. (b) GSCs treated with 1 μM of U73122 for 3 days were subjected to Western blot analyses for the expression of the indicated proteins. (c) Cells transiently treated with 1 μM of U73122 for 3 days were subjected to sphere-forming analyses in the absence of U73122. Right panels show photographs of the representative wells. Graphs show the percentage of wells in which a tumor sphere formed from a single viable cell. Representative images of three biological replicates are shown. Values are presented as the means ± SDs of triplicate samples of an independent experiment. Bars: 100 μm. * $p < 0.05$ vs. Control by the Student's *t*-test.

Supplemental Figure S2

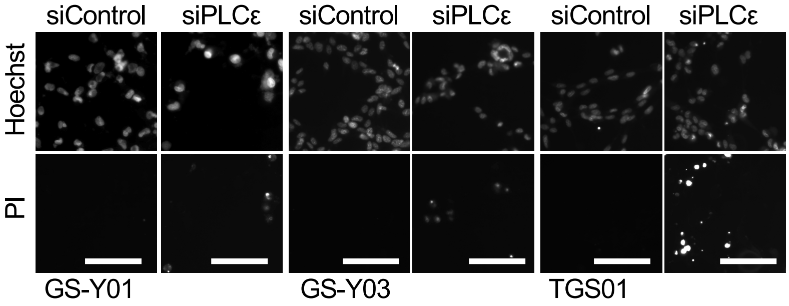

Figure S2. Genetic silencing of PLCε induces cell death. GSCs (GS-Y01, GS-Y03, and TGS01) were transiently transfected with siRNA against PLCE1. Cells were then subjected to PI incorporation analyses. Representative fluorescence images of PI- (lower rows) and Hoechst-positive cells (upper rows) are shown. This figure shows data from the same experiment as Figure 2c. Bars: 100 μm.

Supplemental Figure S3

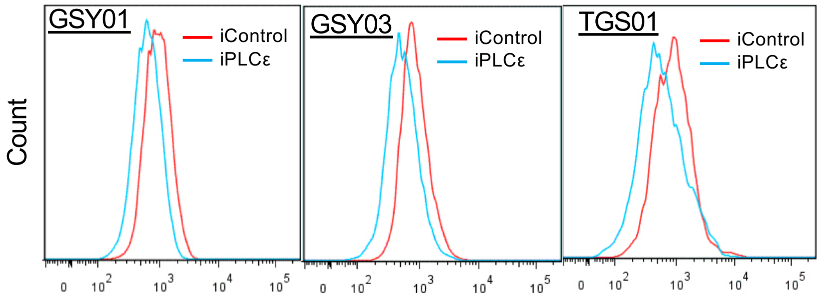

Figure S3. Genetic silencing of PLCε by siRNA suppresses the cell surface expression of CD133. GSCs (GS-Y01, GS-Y03, and TGS01) were transiently transfected with siRNA against PLCE1. Cells were then subjected to flow cytometric analyses. Representative flow cytometric histograms are shown. This figure shows data from the same experiment as Figure 3a.

Supplemental Figure S4

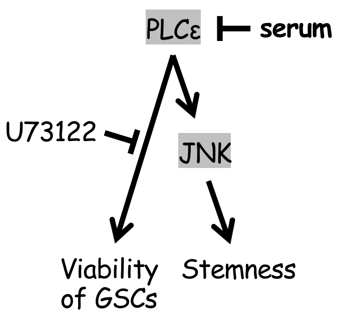

Figure S4. The core scheme of PLCε in the GSCs.
